# GSK461364 Inhibits NLRP3 Inflammasome by Targeting NEK7 Phosphorylation

**DOI:** 10.1002/advs.202504816

**Published:** 2025-09-15

**Authors:** Ruiheng Luo, Mingliang Ma, Dan Wang, Ling Luo, Xueming Xu, Lingmin Huang, Fupeng Wang, Guangyan Kuang, Huiqi Liu, Rui Ni, Xin Li, Qinghua Zhang, Shengfeng Wang, Kai Zhao

**Affiliations:** ^1^ Department of Hematology The Third Xiangya Hospital Central South University Changsha Hunan 410000 P. R. China; ^2^ Department of Dermatology The Third Xiangya Hospital Central South University Changsha Hunan 410000 P. R. China; ^3^ Healthcare Security Department The Affiliated Changsha Central Hospital Hengyang Medical School University of South China Changsha Hunan 410000 P. R. China; ^4^ Key Laboratory of Exploration and Utilization of Aquatic Genetic Resources Ministry of Education Shanghai Ocean University Shanghai 201306 P. R. China; ^5^ China National Pathogen Collection Center for Aquatic Animals Shanghai Ocean University Shanghai 201306 P. R. China; ^6^ Department of Pharmacy The Third Xiangya Hospital Central South University Changsha Hunan 410013 P. R. China; ^7^ Furong Laboratory Central South University Changsha Hunan 410000 P. R. China; ^8^ Key Laboratory of Sepsis Translational Medicine of Hunan Central South University Changsha Hunan 410000 P. R. China

**Keywords:** GSK461364, NEK7, NLRP3 inflammasome, PLK1

## Abstract

NLRP3 inflammasome is a multiple protein complex sensing exogenous or endogenous stimuli, and aberrant activation of the NLRP3 inflammasome is implicated in various inflammatory disorders. While numerous small‐molecule compounds targeting NLRP3 inflammasome activity have been developed, most have encountered limited success in clinical translation. Through screening of a kinase compound library, GSK461364 is identified as a potent and selective NLRP3 inflammasome inhibitor. Notably, GSK461364 confers significant protective effects in murine models of LPS‐induced endotoxemia and DSS‐induced colitis. Mechanistic study reveals that GSK461364 exerts its inhibitory effects via targeting Polo‐like Kinase 1(PLK1). Specifically, that PLK1‐mediated phosphorylation of NEK7, likely occurring at evolutionarily conserved serine residues (Ser221 and Ser260), is shown to enhance NEK7‐NLRP3 binding, a critical step for NLRP3 inflammasome assembly. These findings not only establish GSK461364 as a novel therapeutic candidate for NLRP3‐driven inflammatory diseases but also provide new insights into the regulatory mechanisms governing inflammasome activation through post‐translational modification.

## Graphical abstract

Schematic diagram of the mechanism by which GSK461364 inhibits NLRP3 inflammasome activation. GSK461364 inhibits the phosphorylation of NEK7 likely at serine 221 and serine 260 by suppressing the activity of PLK1, thereby restraining the formation of the NLRP3 inflammasome.

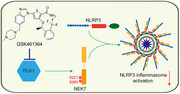

## 1 Introduction

The NLRP3 inflammasome, a multiprotein complex comprising NLRP3, ASC, and caspase‐1, plays a pivotal role in host defense against pathogens and inflammatory responses.^[^ ^1^, ^2^, ^3^ ^]^ Upon exposure to exogenous or endogenous stimuli, such as pathogen‐derived proteins, lipids, nucleic acids, or crystalline materials, the complex assembles, leading to caspase‐1 activation. This process drives the cleavage of pro‐IL‐1β and pro‐IL‐18, as well as gasdermin D‐mediated pyroptotic cell death.^[^ ^1^, ^2^, ^3^, ^4^ ^]^ Aberrant activation of the NLRP3 inflammasome is implicated in diverse inflammatory pathologies, including atherosclerosis, colitis, gout, autoimmune disorders, sepsis, and type 2 diabetes.^[^ ^1^, ^2^, ^3^, ^4^ ^]^ Consequently, targeting the NLRP3 inflammasome represents a promising therapeutic strategy for these conditions.

Although numerous small‐molecule inhibitors targeting NLRP3 or ASC (e.g., MCC950, CY09, tranilast, RRx‐001, oridonin, and MM01) have been developed,^[^ ^5^, ^6^, ^7^, ^8^, ^9^, ^10^ ^]^ most have failed in clinical trials. Recent studies identified NEK7, a NIMA‐related serine/threonine kinase critical for mitotic spindle formation, as an essential NLRP3 inflammasome component.^[^ ^11^, ^12^, ^13^ ^]^ Structural analyses revealed that NEK7 bridges adjacent NLRP3 subunits to facilitate inflammasome activation.^[^ ^14^ ^]^ This specificity positions NEK7 as a compelling therapeutic target. Notably, HT‐6184, a NEK7 inhibitor, recently completed Phase I clinical trials (ClinicalTrials.gov: NCT05447546), underscoring its translational potential. However, additional NEK7‐targeting agents remain underexplored.

Here, through screening a kinase inhibitor library, we identified GSK461364 as a potent inhibitor of NLRP3 inflammasome activation in vitro and in vivo. Mechanistically, GSK461364 targets Polo‐like kinase 1 (PLK1), which phosphorylates NEK7, likely at Ser221 and Ser260, thereby disrupting NLRP3‐NEK7 interaction. These findings position GSK461364 as a promising therapeutic candidate for NLRP3‐related inflammatory diseases.

## 2 Results

### 2.1 Identification of GSK461364 as a Novel NLRP3 Inflammasome Inhibitor via Kinase Library Screening

**Figure 1:**
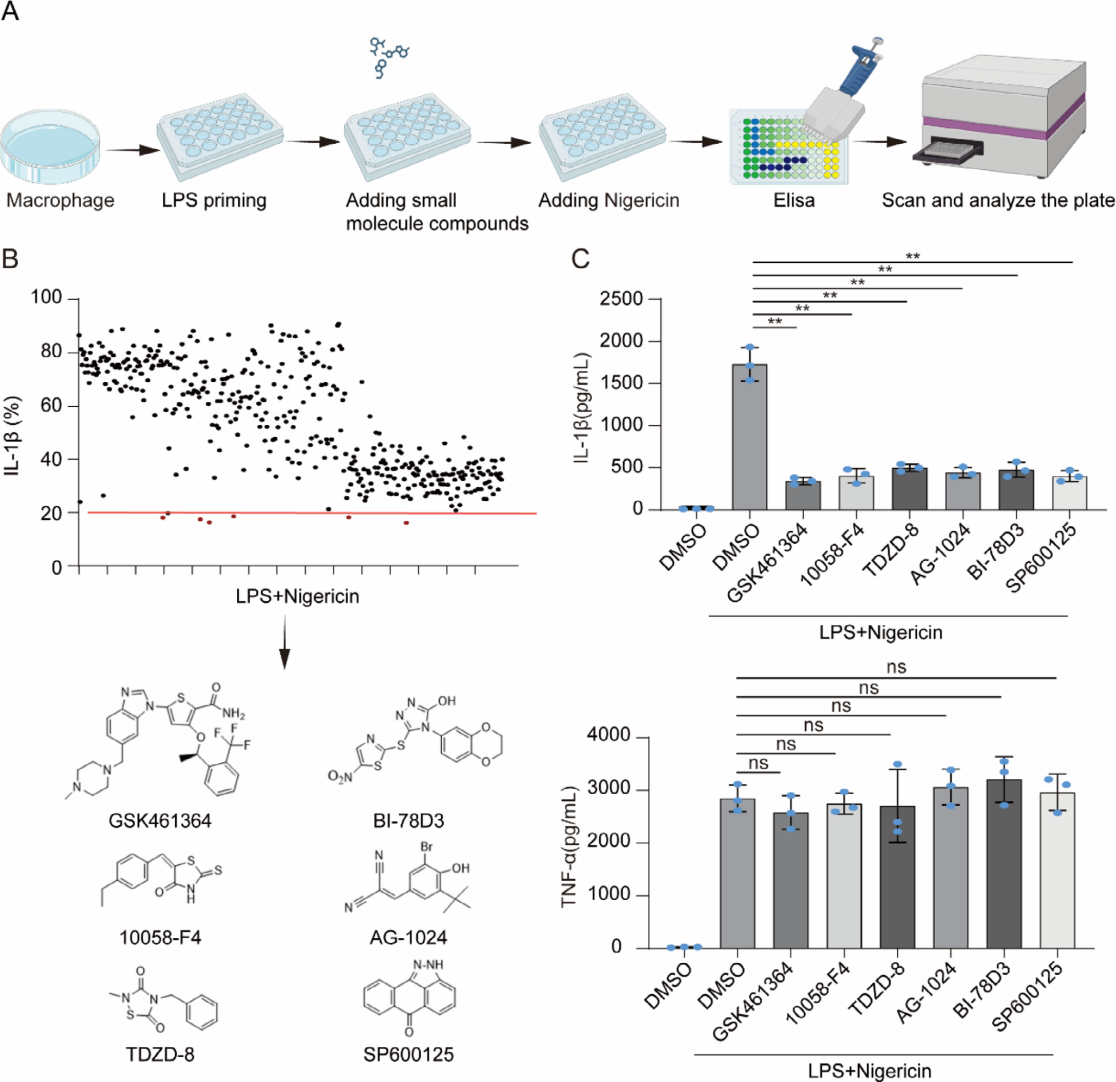
Identification of NLRP3 Inflammasome Inhibitor via Kinase Library Screening. A)Screening of 429 small‐molecule compounds obtained from Selleck Chemicals. Briefly, LPS‐primed macrophages were treated with inhibitors (10 µm) for 1 h, followed by Nigericin treatment. Then, IL‐1β secretion was quantified by ELISA. B)The secretion of IL‐1β by each compound and the chemical structures of the compounds GSK461364, AG‐1024, TDZD‐8, 10058‐F4, SP600125, and BI‐78D. C)ELISA analysis of IL‐1β and TNF‐α secretion in supernatants from LPS‐primed mouse peritoneal macrophages treated with GSK461364, 10058‐F4, TDZD‐8, AG‐1024, BI‐78D, or SP600125, followed by stimulation with nigericin. Results are represented as mean ± SD, and typical photographs are representative of three biological independent experiments with similar results. Statistical analyses were carried out via two‐way ANOVA with the Bonferroni test for(C),^**^ *p* < 0.01.

To identify NLRP3 inflammasome inhibitors, we screened a 429‐compound kinase inhibitor library (Selleck) using an IL‐1β secretion assay induced by NLRP3 agonist (nigericin). To exclude the compounds’ effects on the priming stage, we added these compounds after LPS priming (**Figure** **1**A). Through two screening rounds, six compounds (GSK461364, 10058‐F4, SP600125, BI‐78D3, AG‐1024, and TDZD‐8) were identified to dramatically inhibit IL‐1β secretion without affecting TNF‐α levels (Figure 1B,C). The four compounds (SP600125, BI‐78D3, AG‐1024, TDZD‐8) have been reported to suppress NLRP3 inflammasome by different mechanisms,^[^ ^15^, ^16^, ^17^, ^18^ ^]^ indicating our screening system is able to identify NLRP3 inflammasome inhibitors. GSK461364—a PLK1 inhibitor, merged as a novel candidate.

**Figure 2:**
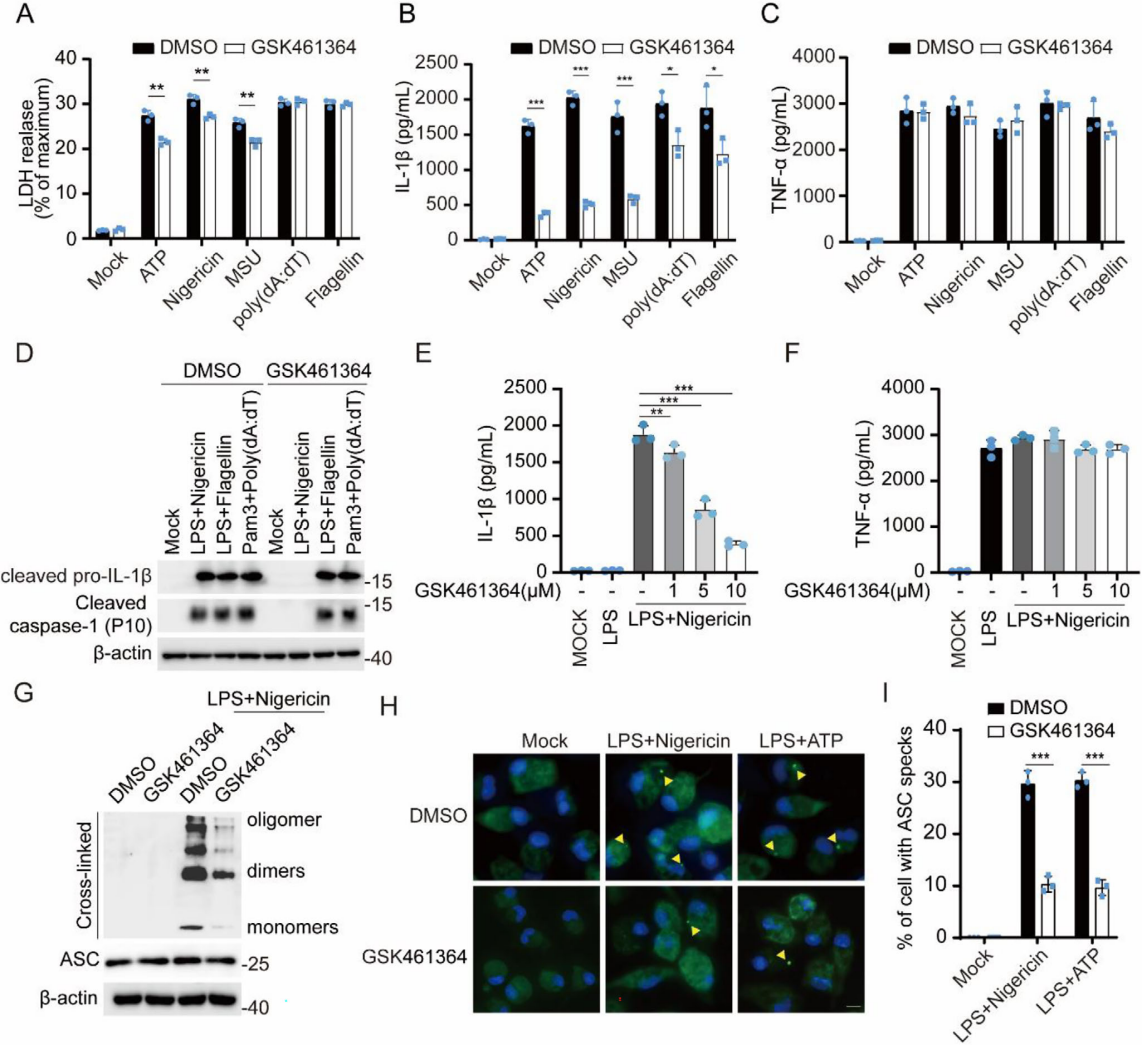
GSK461364 is a specific inhibitor of the NLRP3 inflammasome. A–I)Mouse peritoneal macrophages were first primed with LPS (100 ng mL^−1^, 3 h) and then treated with or without 10 µm GSK461364 for 1 h, followed by stimulation with ATP (5 mm, 1 h), nigericin (10 µm, 1 h), MSU (200 µg mL^−1^, 6 h), flagellin transfection (2 µg mL^−1^, 1 h), or poly(dA:dT) transfection (1 µg mL^−1^, 16 h). A–C)ELISA analysis of IL‐1β and TNF‐α, and release of LDH in supernatants. D)Cell Lysates and supernatant were subjected to western blot analysis. E,F)ELISA analysis of IL‐1β and TNF‐α in supernatants treated with 1 µm, 5 µm, and 10 µm GSK461364. G)Immunoblot analysis of ASC oligomerization in cross‐linked cytosolic pellets treated with DMSO or 10 µm GSK461364. H)Representative immunofluorescence images of ASC specks (green) and nuclei (blue). Yellow arrows indicate ASC specks. Scale bars: 5µm. I)The percentage of cells containing ASC specks was quantified. Results are represented as mean ± SD, and typical photographs are representative of three biological independent experiments with similar results. Statistical analyses were carried out via two‐way ANOVA with the Bonferroni test for(A‐C,E,I)^*^ *p* < 0.05, ^**^ *p* < 0.01 and ^***^ *p* < 0.001.

To further explore the function of GSK461364, we treated LPS‐primed macrophages with kinds of inflammasome agonists: ATP, nigericin, and MSU for the NLRP3 inflammasome, poly(dA:dT) transfection for AIM2 inflammasome, and flagellin for NLRC4 inflammasome. We observed that GSK461364 exhibited a robust inhibition on NLRP3 inflammasome‐induced IL‐1β, but not TNF‐α secretion, as well as cell death (reflected by lactic dehydrogenase (LDH) release) (**Figure** **2**A–C). In contrast, it slightly affected AIM2 or NLRC4 inflammasome‐induced IL‐1β secretion (Figure 2A–C). Accordingly, GSK461364 suppressed NLRP3, but not AIM2 or NLRC4 inflammasome‐induced caspase‐1 cleavage (Figure 2D). Dose‐response experiments revealed maximal inhibition at 10 µm (Figure 2E,F). Additionally, GSK461364 suppressed ASC oligomerization and speck formation, hallmarks of NLRP3 inflammasome assembly (Figure 2G–I). These data establish GSK461364 as a specific NLRP3 inflammasome inhibitor.

### 2.2 Evaluation the Toxicity of GSK461364 Both In Vitro and In Vivo

We further examined the toxicity of GSK461364 in vitro and in vivo. Primary macrophages, HEK293T cells, and iBMDMs were treated with GSK461364 (1–20 µm) for 1 h, followed by 24‐h monitoring. No significant changes in cell viability were observed at any concentration tested(Figure S1A–C, Supporting Information). For the in vivo study, mice were administered 25 mg kg^−1^ GSK461364 and monitored for 9 days. No significant changes in body weight (Figure S1D, Supporting Information), liver function markers (AST/ALT levels) (Figure S1E, Supporting Information) and renal function markers (UREA/CREA levels) (Figure S1F, Supporting Information), Histopathological examination (H&E staining) of major organs (liver, lung, kidney, heart, and spleen) revealed no abnormalities (Figure S1G, Supporting Information). These comprehensive analyses demonstrate that GSK461364 exhibits excellent safety profiles both in vitro and in vivo at the tested concentrations.

### 2.3 GSK461364 Attenuates LPS‐Induced Endotoxemia

**Figure 3:**
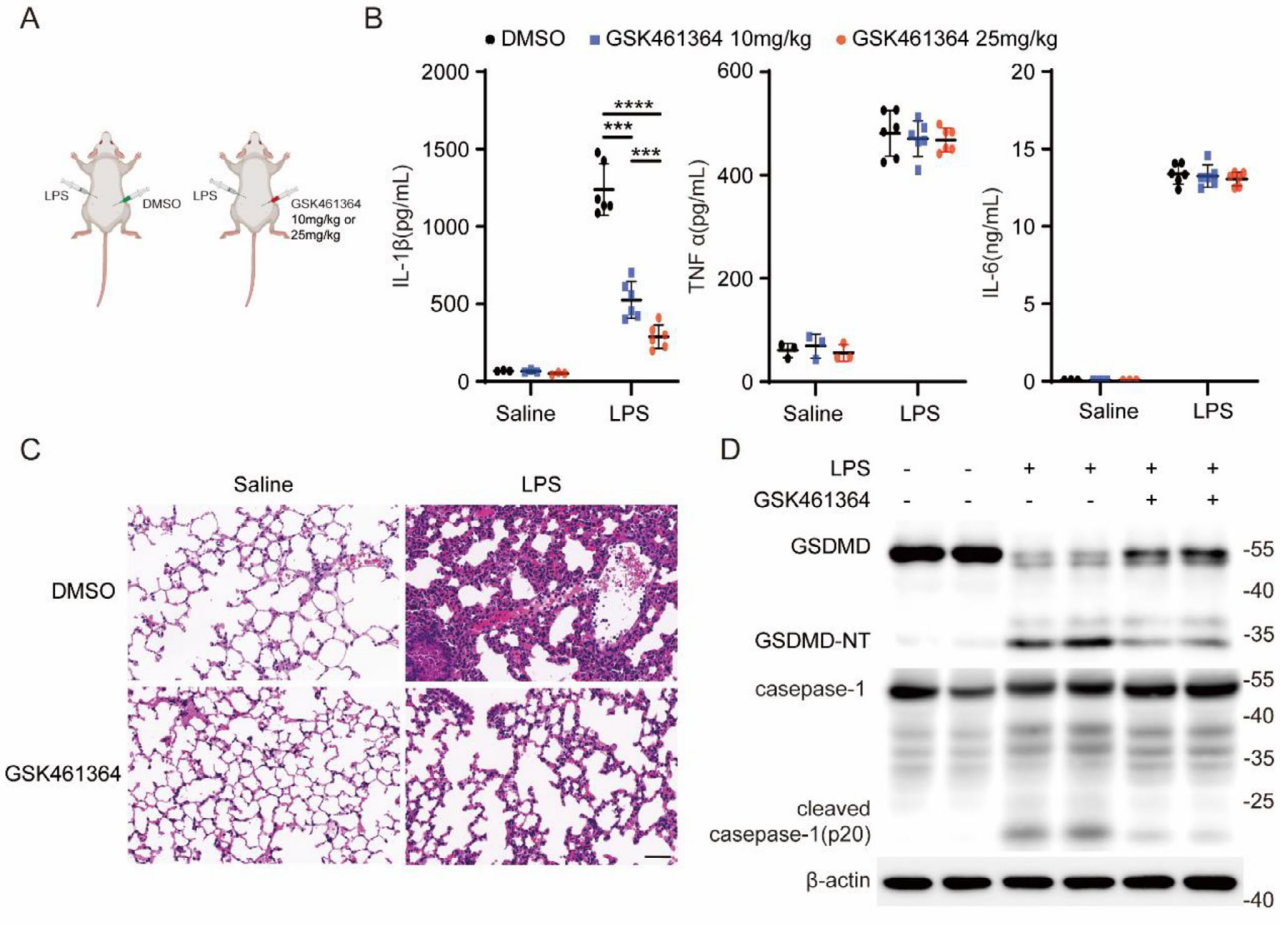
GSK461364 Attenuates LPS‐Induced Endotoxemia. A)chematic representation of the experimental design for LPS and GSK461364 administration in mice. Wild‐type C57BL/6J male mice were administered either DMSO or GSK461364 (10 or 25 mg kg^−1^) intraperitoneally. After 0.5 h, the mice received an injection of either saline or LPS (25 mg kg^−1^, i.p.) (*n* = 6 biologically independent mice) for 12 h. B)ELISA analysis of serum levels of IL‐1β, TNF‐α, and IL‐6 in mice. C)Representative images of hematoxylin and eosin (H&E)‐stained lung tissues of mice treated with GSK461364 (10 mg kg^−1^). Scale bars,50µm. D)Western blot analysis of GSDMD and caspase‐1 in lung tissues of mice treated with GSK461364 (10 mg kg^−1^). Results are represented as mean ± SD, and typical photographs are representative of three biological independent experiments with similar results. Statistical analyses were carried out via two‐way ANOVA with the Bonferroni test for(B), ^***^ *p* < 0.001.

Next, to evaluate in vivo efficacy, we employed an LPS‐induced endotoxemia model. Mice were intraperitoneally injected with two doses of GSK461364 (10 and 25 mg kg^−1^) before LPS (25 mg kg^−1^) treatment (**Figure** **3**A). GSK461364 pretreatment group showed dose‐dependent reductions in serum IL‐1β (but not TNF‐α or IL‐6) (Figure 3B) and diminished lung inflammation, as evidenced by histopathology (Figure 3C) and reduced GSDMD cleavage (GSDMD‐NT) and caspase‐1 cleavage (p20) (Figure 3D), compared to that of the control group.

### 2.4 GSK461364 Ameliorates DSS‐Induced Colitis

**Figure 4:**
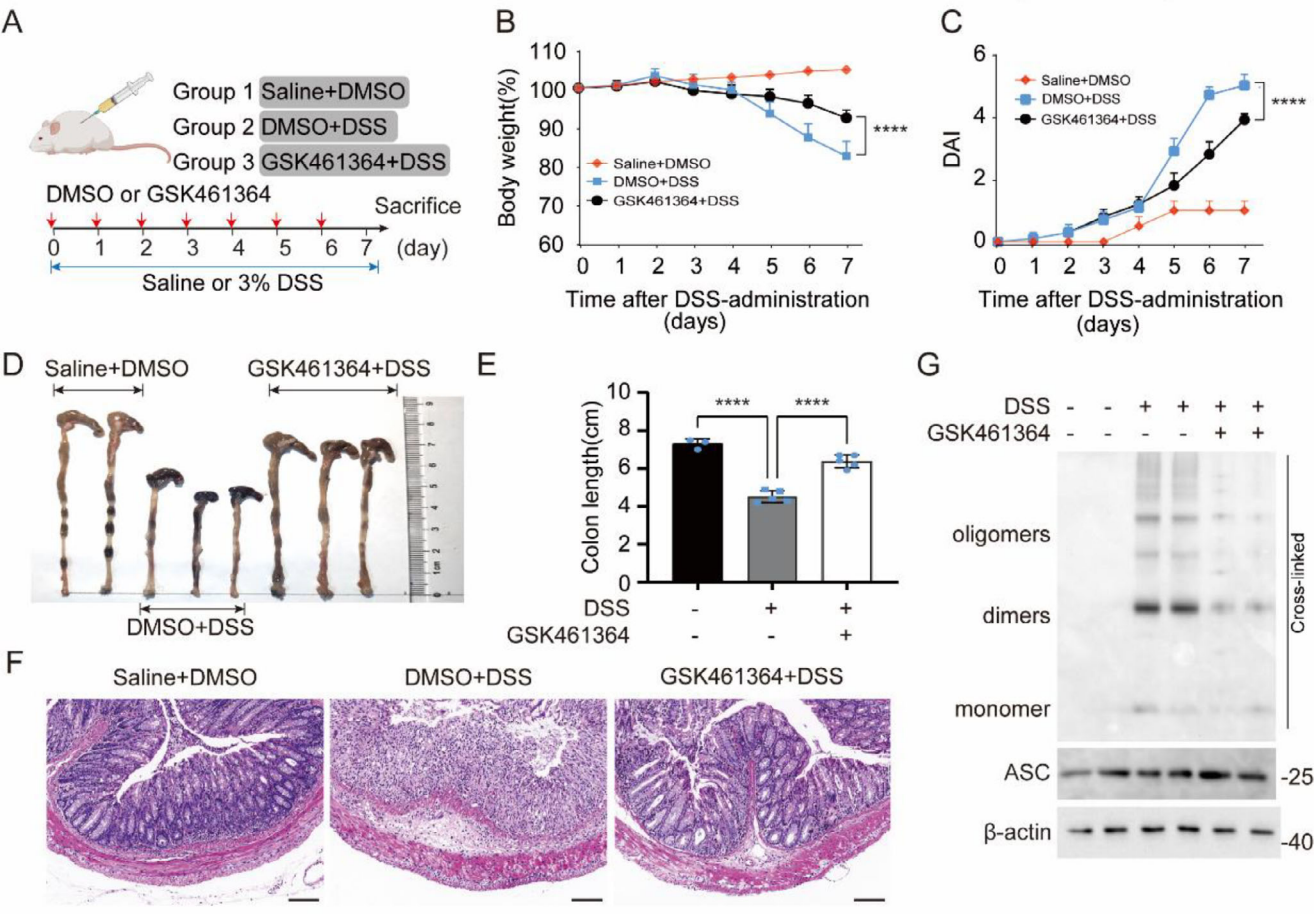
GSK461364 Ameliorates DSS‐induced Colitis. A)Wild‐type mice were administered either 3% DSS in drinking water or normal drinking water for a duration of 7 days. Concurrently, GSK461364 was administered via intraperitoneal injection once daily from day 0 to day 7. B)Body weights of mice were recorded daily. C)DAI scores of mice were assessed and recorded daily. D) Colon lengths of mice. E)The average colon length of mice was calculated. F)HE staining of colon tissues. Scale bars,100µm. G)Immunoblot analysis of ASC oligomerization in colon tissues. Results are represented as mean ± SD, and typical photographs are representative of three biological independent experiments with similar results. Statistical analyses were carried out via two‐way ANOVA with the Bonferroni test for(B,C,E), ^***^ *p* < 0.001.

Dysregulated NLRP3 inflammasome contributes to the pathogenesis of colitis,^[^ ^19^ ^]^ we then employed a dextran sulfate sodium (DSS)‐induced acute colitis model. Mice were challenged with 3% DSS for 7 days, and GSK461364 was intraperitoneally injected daily from day 0 to day 7 (**Figure** **4**A). In comparison with the DSS‐treated group, GSK461364 administration mitigated weight loss, disease activity index (DAI) scores, colon shortening, and histopathological damage (Figure 4B–F). Furthermore, ASC oligomerization in colon tissues—a marker of NLRP3 inflammasome activation—was significantly reduced (Figure 4G). Taken together, these data demonstrated that GSK461364 could alleviate the NLRP3‐related inflammatory responses in vivo.

### 2.5 GSK461364 Targets PLK1 for the Inhibition of NLRP3 Inflammasome

Next, we explored the underlying mechanism for GSK461364 suppressing the NLRP3 inflammasome. First, we detected whether GSK461364 affected the well‐known upstream pathways of NLRP3 inflammasome,^[^ ^3^, ^20^ ^]^ including mitochondrial injury (Figure S2A, Supporting Information), mitochondrial ROS production (Figure S2B, Supporting Information), potassium efflux (Figure S2C, Supporting Information), and chlorine efflux (Figure S2D, Supporting Information), the results showed that GSK461364 treatment did not alter any of these key NLRP3 regulatory pathways (Figure S2A–D, Supporting Information). Furthermore, the mRNA expression of NLRP3 andIL‐1β, and the protein expression of NLRP3, ASC, Caspase‐1, NEK7, as well as pro‐IL‐1β, were not influenced by GSK461364 treatment (Figure S2E,F, Supporting Information). Thus, GSK461364 likely inhibits NLRP3 inflammasome without directly targeting NLRP3 and its associated pathways.

**Figure 5:**
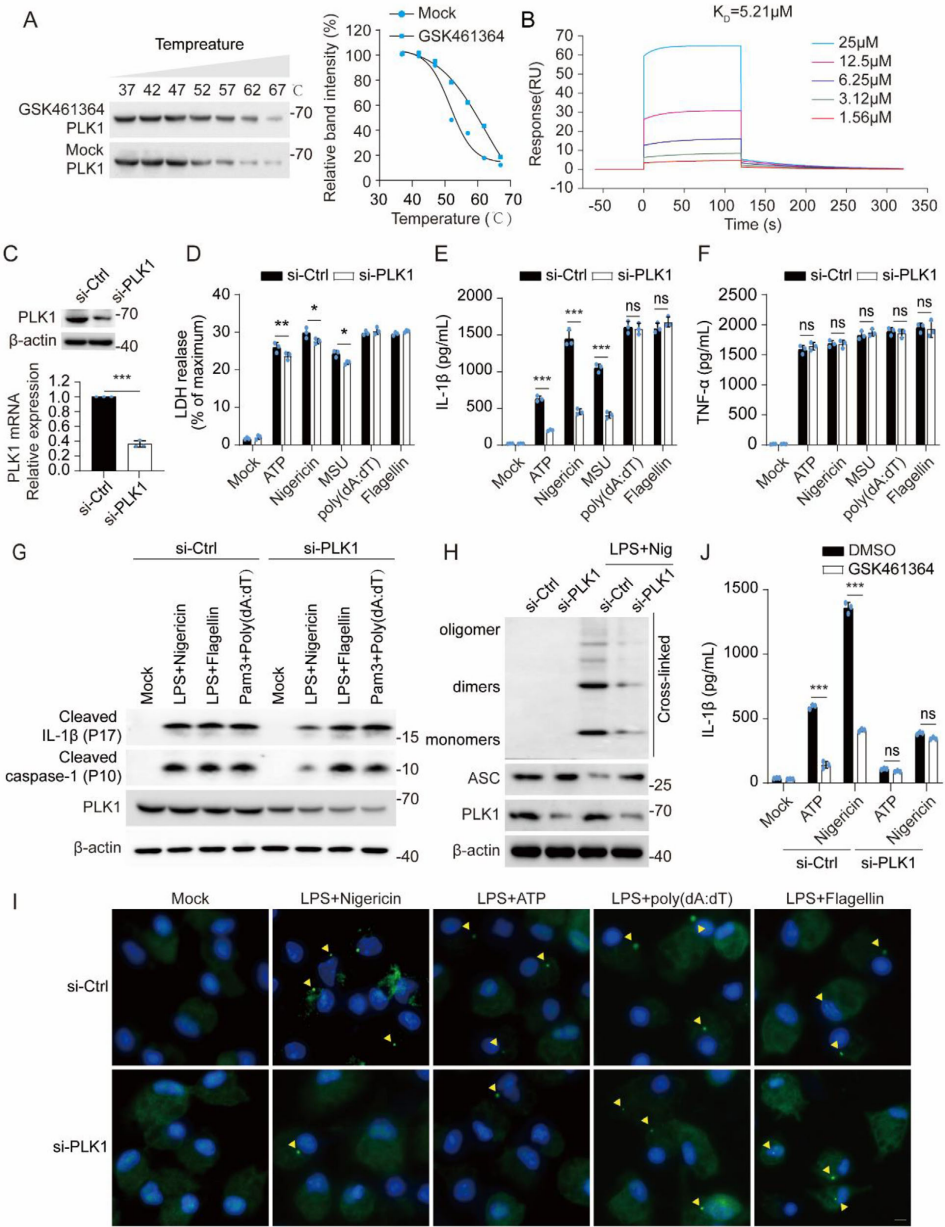
GSK461364 targets PLK1 for the inhibition of NLRP3 inflammasome. A)Western blot analysis of Cellular Thermal Shift Assay (CETSA) for PLK1 binding to GSK461364 (10 µm) and the melting curve generated from CETSA was analyzed using ImageJ software. B)Surface plasmon resonance (SPR) analysis of the interaction between PLK1 and GSK461364. C)Western blot analysis and quantitative real‐time PCR analysis of PLK1 expression in mouse peritoneal macrophages transfected with si‐Ctrl or si‐PLK1 for 48 h. D–L)Mouse peritoneal macrophages were transfected with si‐Ctrl or si‐PLK1 for 48 h, primed with LPS (100 ng mL^−1^, 3 h), and subsequently stimulated with ATP (5 mm, 1 h), nigericin (10 µm, 1 h), MSU (200 µg mL^−1^, 6 h), flagellin (2 µg mL^−1^, 1 h, transfection), or poly(dA:dT) (1 µg mL^−1^, 16 h, transfection). D‐F)ELISA analysis of IL‐1 β and TNF‐α, and release of LDH in supernatants. G)Cell lysates and supernatants were analyzed by Western blot. H)Immunoblot analysis of ASC oligomerization in cross‐linked cytosolic pellets. I)Representative immunofluorescence images of ASC specks (green) and nuclei (blue). Yellow arrows indicate ASC specks. Scale bars: 5µm. J–L)ELISA analysis of IL‐1β and TNF‐α, and release of LDH in supernatants from mouse peritoneal macrophages treated with DMSO or GSK461364 (1 h) after si‐Ctrl or si‐PLK1 transfection for 48h. Results are represented as mean ± SD, and typical photographs are representative of three biological independent experiments with similar results. Statistical analyses were carried out via two‐way ANOVA with the Bonferroni test for(C‐E), ^*^ *p* < 0.05, ^**^ *p* < 0.01 and ^***^ *p* < 0.001.

Given that PLK1 is the target of GSK461364, we decided to investigate the role of PLK1 in NLRP3 inflammasome activation. GSK461364 had no effect on the expression of PLK1(Figure S2E,F, Supporting Information); however, it could suppress the kinase activity of PLK1.^[^ ^21^, ^22^, ^23^ ^]^ By using the cellular thermal shift assay and surface plasmon resonance (SPR) assay, we confirmed GSK461364 binds to PLK1 with an estimated *K_D_* of 5.21 µm (**Figure** **5**A,B), making us perform the following experiments. Then, we silenced PLK1 in primary macrophages (Figure 5C), and treated macrophages with a series of NLRP3 agonists: ATP, nigericin, and MSU, NLRC4 agonist: flagellin, and AIM2 agonist: poly(dA:dT). Akin to the effect of GSK461364, silence of PLK1 impaired NLRP3, but not NLRC4 or AIM2 inflammasome‐induced cell death, IL‐1β secretion, and caspase‐1 maturation (Figure 5D–G). Moreover, ASC oligomerization and speck formation were both inhibited by the silencing PLK1 (Figure 5H,I). To definitively confirm whether GSK461364 inhibits NLRP3 inflammasome activation via PLK1, we treated wild‐type and PLK1‐silenced primary macrophages with GSK461364. We observed that the robust inhibitory effect of GSK461364 on IL‐1β release in wild‐type macrophages was lost in PLK1‐silenced macrophages (Figure 5J). Thus, GSK461364 suppresses the NLRP3 inflammasome activation through targeting PLK1.

### 2.6 PLK1 Promotes the Association Between NLRP3 and NEK7

**Figure 6:**
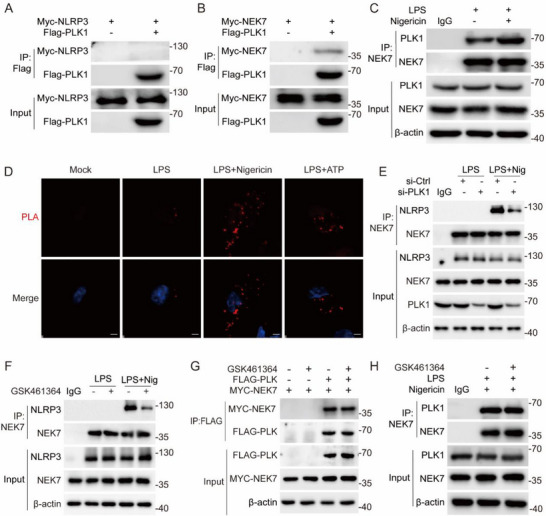
PLK1 promotes the association between NLRP3 and NEK7. A) Co‐immunoprecipitation and immunoblot analysis of lysates from HEK293T cells transfected with Flag‐PLK1 and Myc‐NLRP3. Immunoprecipitation was performed using anti‐Flag and anti‐Myc antibodies. B) Co‐immunoprecipitation and immunoblot analysis of lysates from HEK293T cells transfected with Flag‐PLK1 and Myc‐NEK7. Immunoprecipitation was performed using anti‐Flag and anti‐Myc antibodies. C)Co‐immunoprecipitation and immunoblot analysis of lysates from mouse peritoneal macrophages treated with LPS (100 ng mL^−1^, 3 h) followed by Nigericin (10 µm, 1 h) or untreated. D)The physical interaction between PLK1 and NEK7 was visualized as red puncta using proximity ligation assay (PLA) in mouse peritoneal macrophages primed with LPS (100 ng mL^−1^, 3 h) and subsequently stimulated with nigericin (10 µm, 1 h) and ATP (100 µm, 1 h) for 1 h. Scale bars: 1 µm. E)Co‐immunoprecipitation and immunoblot analysis of lysates from mouse peritoneal macrophages treated with si‐Ctrl or si‐PLK1 followed by LPS (100 ng mL^−1^, 3 h), or subsequently stimulated with nigericin (10 µm, 1 h). F)Co‐immunoprecipitation and immunoblot analysis of lysates from mouse peritoneal macrophages treated with LPS (100 ng mL^−1^, 3 h) followed with or without GSK461364, or subsequently stimulated with nigericin (10 µm, 1 h). G)Co‐immunoprecipitation and immunoblot analysis of lysates from HEK293T cells transfected with Flag‐PLK1 and Myc‐NEK7 treated with or without 10 µm GSK461364 for 1 h. Immunoprecipitation was performed using anti‐Flag and anti‐Myc antibodies. H)Co‐immunoprecipitation and immunoblot analysis were performed on lysates from mouse peritoneal macrophages primed with LPS (100 ng mL^−1^, 3 h), treated with or without 10 µm GSK461364 for 1 h, and subsequently stimulated with nigericin (10 µm, 1 h).

To elucidate the molecular mechanism underlying PLK1‐mediated NLRP3 inflammasome activation, we first investigated the protein interaction network. Co‐immunoprecipitation assays in both HEK293T cells and macrophages revealed specific interaction between PLK1 and NEK7, but not with NLRP3 (**Figure** **6**A–C). This physical association was further confirmed by proximity ligation assay (PLA), demonstrating in situ complex formation between PLK1 and NEK7 (Figure 6D). Given the established importance of NEK7‐NLRP3 interaction in inflammasome assembly,^[^ ^14^ ^]^ we next examined the regulatory role of PLK1 in this process. Remarkably, both PLK1 silencing and pharmacological inhibition using GSK461364 significantly impaired NEK7‐NLRP3 complex formation, as evidenced by co‐immunoprecipitation analysis (Figure 6E,F). However, GSK461364 had no effect on the interaction between PLK1 and NEK7 in both HEK293T cells and primary macrophages (Figure 6G,H). These findings collectively demonstrate that PLK1 serves as a critical regulator of NEK7‐NLRP3 interaction.

### 2.7 PLK1 Mediates NEK7 Phosphorylation, Likely at Ser221 and Ser260

**Figure 7:**
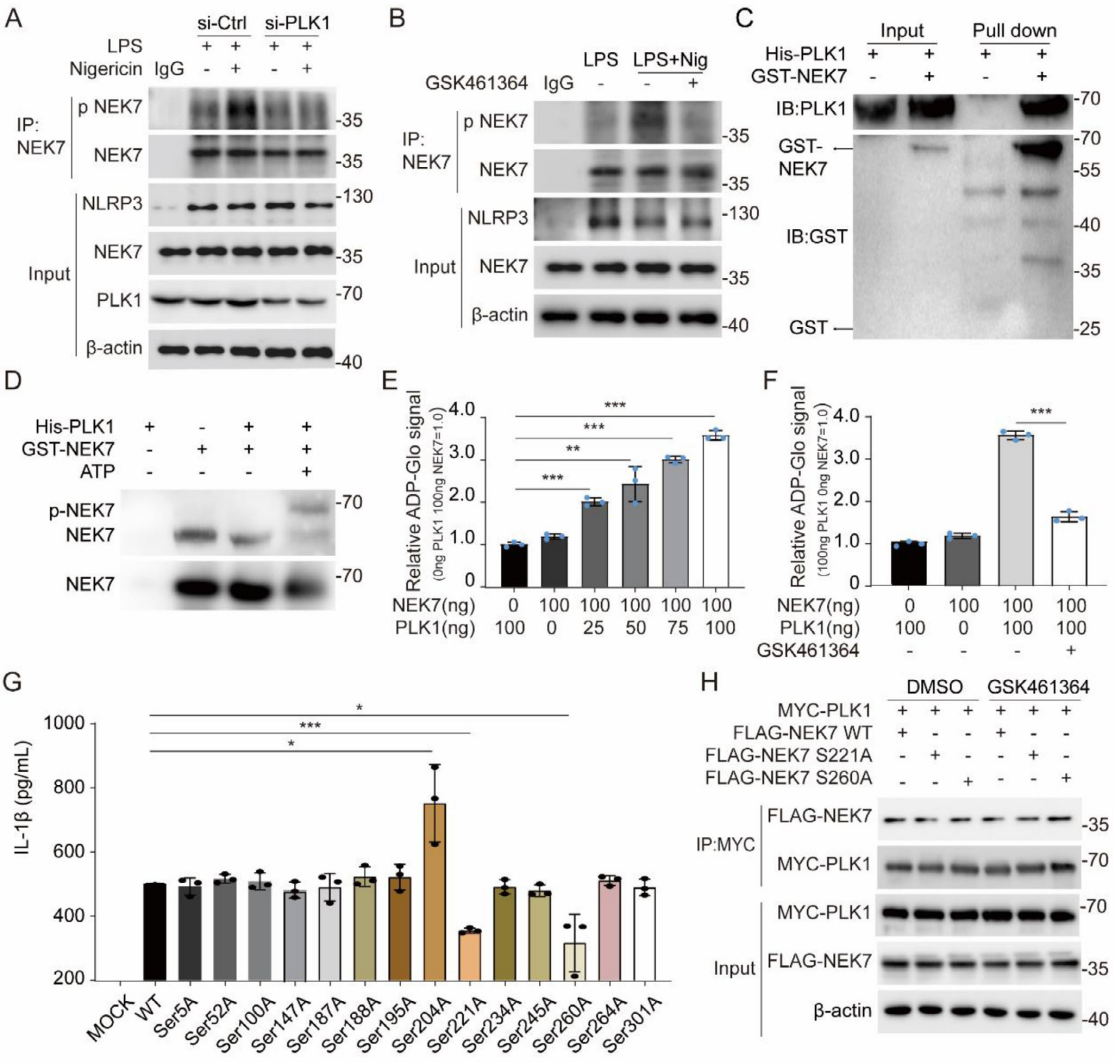
PLK1 mediates NEK7 phosphorylation, likely at Ser221 and Ser260. A)Immunoprecipitation and immunoblotting analysis of lysates from mouse peritoneal macrophages transfected with si‐Ctrl or si‐PLK1, primed with LPS (100 ng mL^−1^, 3 h), and subsequently stimulated with or without nigericin (10 µm, 1 h), using anti‐NEK7 antibody and anti‐pan phospho‐serine/threonine antibody. B)Immunoprecipitation and immunoblotting analysis of lysates from mouse peritoneal macrophages primed with LPS (100 ng mL^−1^, 3 h), treated with or without 10 µm GSK461364 for 1 h, and subsequently stimulated with or without nigericin (10 µm, 1 h), using anti‐NEK7 antibody and anti‐pan phospho‐serine/threonine antibody. C)GST pull‐down analysis of the interaction between GST‐NEK7 and His‐PLK1. D)Immunoblot analysis of NEK7 by SDS‐PAGE and Phos‐tag SDS‐PAGE. E)ADP‐Glo quantification of kinase activity at different concentrations of PLK1. F)ADP‐Glo quantification of kinase activity with DMSO or 10 µm GSK461364. G)The levels of IL‐1β released into the supernatant of 293T cells were measured following transfection with plasmids encoding IL‐1β, caspase‐1, ASC, NLRP3, PLK1, NEK7 WT, and NEK7 mutant, and subsequent stimulation with nigericin. H)Co‐immunoprecipitation and immunoblot analysis of lysates from HEK 293T cells transfected with Myc‐PLK1 and Flag‐NEK7 WT, Flag‐NEK7 Ser221, or Flag‐NEK7 Ser260. Immunoprecipitation was performed using anti‐Flag and anti‐Myc antibodies. Results are represented as mean ± SD, and typical photographs are representative of three biological independent experiments with similar results. Statistical analyses were carried out via two‐way ANOVA with the Bonferroni test for(E), ^*^ *p* < 0.05, ^**^ *p* < 0.01, and ^***^ *p* < 0.001.

Given the kinase‐dependent nature of PLK1's action, we hypothesized that PLK1 catalytic activity regulates NLRP3‐NEK7 association through phosphorylation. Immunoprecipitation‐phosphorylation profiling showed that nigericin‐induced Ser/Thr phosphorylation of NEK7 was substantially attenuated upon PLK1 knockdown or GSK461364 treatment (**Figure** **7**A,B), establishing PLK1 as a key mediator of NEK7 phosphorylation. GST pull‐down assays using purified proteins confirmed direct PLK1‐NEK7 binding (Figure 7C), providing a structural basis for the enzymatic reaction.

To demonstrate direct phosphorylation, we performed Phos‐tag SDS‐PAGE analysis, which allows phosphorylated proteins to be detected by Western blotting without the need for phospho‐specific antibodies,^[^ ^24^ ^]^ and ADP‐Glo Kinase Assay analysis, which was used to evaluate the activity of the kinase or phosphatase.^[^ ^25^ ^]^ The appearance of retarded migration bands specifically in PLK1‐containing reactions (Figure 7D), and the increased ADP generation signal in the PLK1‐added group in a dose‐dependent manner (Figure 7E), provided conclusive evidence for PLK1‐dependent NEK7 phosphorylation. Additionally, GSK461364 significantly reduced the extent of NEK7 phosphorylation mediated by PLK1 (Figure 7F), further proving that GSK461364 targets PLK1's kinase activity.

Then, to figure out the phosphorylation site in NEK7, the NetPhos 3.1 server (https://services.healthtech.dtu.dk/services/NetPhos‐3.1/) was used, and 14 Ser/Thr phosphorylation sites in NEK7 were predicted. We replaced all the Ser/Thr with Ala to mimic the non‐phosphorylated state, and found that when we introduced NEK7‐S221A or NEK7‐S260A into HEK293T cells that were reconstituted with NLRP3 inflammasome.^[^ ^26^ ^]^ IL‐1β secretion was impaired, compared to the NEK7 wild type; the positive control mutant NEK7‐S204A showed enhanced IL‐1β production as previously reported^[^ ^27^ ^]^ (Figure 7G). Notably, S221A or S260A mutation in NEK7 did not affect the interaction between NEK7 and PLK1 in both the presence and absence of GSK461364 (Figure 7H).

These data collectively established that PLK1 may phosphorylate NEK7 at Ser221/Ser260 to promote NLRP3 inflammasome activation.

## 3 Discussion

Through systematic screening of a customized kinase inhibitor library, we identified GSK461364 as a potent inhibitor of the NLRP3 inflammasome. Pharmacological validation demonstrated its protective effects in both LPS‐induced endotoxemia and DSS‐induced colitis models. Mechanistic investigations revealed that GSK461364 suppresses NLRP3 inflammasome assembly by inhibiting PLK1 kinase activity. This inhibition abrogates NEK7 phosphorylation, thereby disrupting its interaction with NLRP3. Our findings position GSK461364 as a promising therapeutic candidate for NLRP3‐driven disorders.

Current NLRP3 inhibitors (e.g., MCC950, CY09, tranilast, RRx‐001, and oridonin) primarily target NLRP3 itself through direct binding, yet their clinical translation remains challenging despite preclinical efficacy.^[^ ^6^, ^7^, ^8^, ^9^, ^10^ ^]^ Emerging strategies focus on NEK7, an essential component for NLRP3 inflammasome assembly.^[^ ^11^, ^12^ ^]^ Recent advances include entrectinib (FDA‐approved anticancer agent), which disrupts NLRP3‐NEK7 interaction via NEK7 binding,^[^ ^28^ ^]^ and HT‐6184, currently in phase I trials (NCT05447546). Our study extends this paradigm by demonstrating that GSK461364‐mediated suppression of NEK7 phosphorylation effectively blocks NLRP3 inflammasome activation. This aligns with emerging evidence highlighting post‐translational modification (PTM) regulation of NEK7 (e.g., deglutathionylation^[^ ^29^ ^]^) as a viable therapeutic strategy. However, the potential roles of other NEK7 PTMs (acetylation, methylation) and corresponding modulators require further exploration.

While GSK461364 exhibits established antitumor activity across multiple malignancies (e.g., lung carcinoma, breast cancer, hepatocellular carcinoma, osteosarcoma, and glioblastoma)^[^ ^30^, ^31^, ^32^, ^33^, ^34^, ^35^ ^]^ and has completed phase I evaluation,^[^ ^36^ ^]^ our study reveals its novel anti‐inflammatory properties via NLRP3 suppression. Given the established link between chronic inflammation and carcinogenesis (e.g., hepatitis‐associated hepatocellular carcinoma, colitis‐associated colorectal cancer), GSK461364 may offer dual therapeutic benefits in inflammation‐driven malignancies.

Our work establishes PLK1, a serine/threonine kinase critical for mitotic progression,^[^ ^37^ ^]^ as a novel regulator of NLRP3 inflammasome activation. Beyond its canonical role in cell cycle regulation, PLK1 has been involved in a series of biological processes, including centrosome biology, spindle dynamics, chromosome segregation, and cytokinesis, via phosphorylating different substrates.^[^ ^37^ ^]^ In this study, we identified PLK1‐mediated phosphorylation of NEK7, likely at Ser221/Ser260, as a critical driver of NLRP3 inflammasome assembly. This contrasts with recent findings^[^ ^38^ ^]^ suggesting PLK1 modulates NLRP3 spatial organization via PLK1‐NLRP3 interaction, highlighting the complexity of PLK1's role in inflammasome regulation. We think the difference may come from that the NLRP3 inflammasome is a huge complex, and the immunoprecipitation assay could not precisely distinguish. However, we both observed that the kinase activity of PLK1 is involved in the regulation of the NLRP3 inflammasome.

Notably, divergent effects within the Polo‐like kinase family are observed: PLK4 has been reported to suppress the NLRP3 inflammasome through NEK7 Ser204 phosphorylation. This observation that NEK7 phosphorylation seems to be contrary to our conclusion. The discrepancy may be due to diverse phosphorylated sites at NEK7 play different roles, which is similar to the phosphorylation that occurs at NLRP3, where Ser5 phosphorylation inhibits NLRP3 activation, while Ser194 phosphorylation promotes it.^[^ ^17^, ^39^ ^]^ Furthermore, besides in the regulation of the NLRP3 inflammasome, the function of PLK1 and PLK4 in tumorigenesis is opposite, since PLK1 displays some oncogenic activity, while PLK4 functions as a tumor suppressor.^[^ ^40^ ^]^ These findings suggest phosphorylation site specificity critically determines functional outcomes, emphasizing the need for precise kinase targeting strategies.

In conclusion, our study delineates a novel PLK1/NEK7/NLRP3 axis in inflammasome regulation, with GSK461364 emerging as a dual‐function inhibitor suppressing phosphorylation of NEK7 and subsequent inflammasome assembly. The compound demonstrated significant efficacy in endotoxemia and colitis models, supporting its therapeutic potential for NLRP3‐mediated disorders. These findings advance our understanding of kinase network regulation in innate immunity and provide a molecular rationale for repurposing PLK1 inhibitors in inflammatory diseases.

## 4 Experimental Section

### Mice

Wild‐type C57BL/6 mice (8 weeks old) were purchased from Hunan SJA Laboratory Animal Co., Ltd (Changsha, China). Animals were maintained in a specific pathogen‐free environment at the Department of Laboratory Animals of Central South University. All experimental animal protocols were approved by the Institutional Animal Care and Use Committees of Central South University (APU‐2025‐0019).

### Reagents

ATP (tlrl‐atpl), Ultra‐pure LPS (for cell, tlrl‐peklps), LPS(for mice, tlrl‐eklps), MSU (tlrl‐msu), Nigericin (tlrl‐nig), Pam3CSK4 (tlrl‐pms), FLA‐ST (tlrl‐stfla), and Poly(dA:dT) (tlrl‐patn) were purchased from Invivogen. Anti‐NLRP3 antibody (AdipoGen, AG‐20B‐0014‐C100) and anti‐ASC antibody (AdipoGen, AG‐25B‐0006‐C100), anti‐Caspase‐1 antibody(Abcam, ab179515), anti‐NEK7 antibody(Abcam, ab133514), anti‐IL‐1β antibody (RD Systems, AF‐401‐NA), anti ‐PLK1 antibody(ABclonal, A2548), anti‐β‐actin antibody (Cell Signaling Technology, #3700), anti‐DDDDK‐tag antibody(Cell Signaling Technology, #2368), anti‐Myc‐tag antibody(Cell Signaling Technology, #2272), anti ‐His‐tag antibody(MBL, d291‐3) were obtained from the indicated suppliers. Kinase inhibitor library (L1200) and GSK461364 (S2193) were from Selleck Chemicals.

### Kinase Inhibitor Screening Assay

Seed peritoneal macrophages in 96‐well plates at a density of 30 000 cells per well. Pre‐stimulate cells with 100 ng mL^−1^ LPS for 3 h. Add kinase inhibitors (final concentration: 10 µm) and incubate for 1h. Subsequently, treat with 10 µm Nigericin for 1h. Kinase Inhibitor Library (L1200) was purchased from Selleck Chemicals.

### Intracellular Ion Concentration Analysis

Following treatment with specified stimuli, supernatants were carefully aspirated. Peritoneal macrophages were subsequently washed twice with PBS. Intracellular potassium (K⁺) and chloride (Cl^−^) concentrations were determined using assay kits (Potassium Assay Kit, Cat# C001‐2‐1, Chlorine Assay Kit, Cat# C003‐2‐1, Nanjing Jiancheng Bioengineering Institute, China) according to the manufacturer's protocols.

### Analysis of Mitochondrial ROS and Membrane Potential

Peritoneal macrophages were seeded in 6‐well plates and treated with the specified stimuli according to the experimental design. After treatment, cells were incubated with pre‐diluted MitoSOX Red(final concentration: 500 nm) or TMRM (final concentration: 200 nm) in a humidified 37 °C, 5% CO_2_ incubator for 30 min. After incubation, gently wash the cells twice with PBS and observe mitochondrial ROS and membrane potential levels using a red fluorescence filter.

### Quantitative PCR

Total RNA was extracted using the RNA Fast 200 kit (FASTAGEN, China) following the manufacturer's instructions. Complementary DNA was performed with the TransScript All‐in‐One First‐Strand cDNA Synthesis SuperMix for qPCR (TransGen Biotech, China) according to the manufacturer's instructions. Quantitative PCR was performed using SYBR Green (Vazyme Biotech) on a LightCycler 480 (Roche Diagnostics). Gene expression levels were normalized to β‐actin as an internal control, and relative expression changes were calculated using the 2−ΔΔCT method. The primers were as follows: NEK7 forward: 5′‐AAAGAGGCTAATCCCTGAGAGAA‐3′, NEK7 reverse: 5′‐CTACCCCAGTGGCTGTAATGA‐3′.PLK1 forward: 5′‐CTTCGCCAAATGCTTCGAGAT‐3′, PLK1 reverse: 5′‐TAGGCTGCGGTGAATTGAGAT‐3′.IL‐1β forward: 5′‐GAAATGCCACCTTTTGACAGTG‐3′, IL‐1β reverse: 5′‐TGGATGCTCTCATCAGGACAG‐3′.NLRP3 forward: 5′‐TGGATGGGTTTGCTGGGA‐3′, NLRP3 reverse: 5′‐ CTGCGTGTAGCGACTGTTGAG‐3′.β‐actin forward: 5′‐ AGTGTGACGTTGACATCCGT‐3′, β‐actin reverse: 5′‐GCAGCTCAGTAACAGTCCGC‐3′.

### In Vitro PLK1 Kinase Assay

The kinase reaction was performed by incubating 100 ng of mouse recombinant PLK1 (Sino Biological, China) with 100 ng of NEK7 substrate (Sino Biological, China) in 1× reaction buffer (40 mm Tris‐HCl, pH 7.5, 20 mm MgCl_2_;0.1 mg mL^−1^ BSA) containing 10 µm ATP. After a 60‐min incubation at room temperature, the reaction was terminated, and the generated ADP was quantified using the ADP‐Glo Kinase Assay System (Promega, USA) according to the manufacturer's instructions. Briefly, 10 µL of ADP‐Glo reagent was added to the 10 µL reaction mixture and incubated for 40 min at room temperature. Subsequently, 20 µL of kinase detection reagent was added, followed by an additional 30‐min incubation. Luminescence signals were measured using a Varioskan Flash multimode reader (Thermo Fisher Scientific). Data were presented as relative luminescence units.

### Toxicity Assessment of GSK461364

Target cells were plated in 96‐well plates and exposed to a concentration gradient of GSK461364 or DMSO for 1 h under standard culture conditions (37 °C, 5% CO_2_). Cell viability was subsequently determined using the CellTiter‐Glo Luminescent Cell Viability Assay (Promega, USA) following the manufacturer's protocol. C57BL/6 mice were randomly divided into two groups:(DMSO vehicle control group, *n* = 3, GSK461364 treatment group, *n* = 3). Both treatments were administered via intraperitoneal injection. Body weight was monitored daily at consistent time points throughout the 9‐day study period. Tissue samples for hematoxylin and eosin (H&E) staining, and terminal serum samples were collected and submitted to Wuhan Servicebio Technology Co., Ltd for analysis.

### Macrophage Cultures and Stimulation

Mouse primary peritoneal macrophages cells were harvested and planted as previously described.^[^ ^41^ ^]^ For NLRP3 inflammasome activation, macrophages were primed with ultra‐pure LPS (100 ng mL^−1^) for 3 h and stimulated with ATP (5 mm, 1 h), Nigericin (10 µm, 1 h), or MSU (200 µg mL^−1^, 6 h). For NLRC4 inflammasome activation, macrophages were primed with ultra‐pure LPS (100 ng mL^−1^) for 3 h and transfected with flagellin (2 µg mL^−1^) by Lipofectamine 3000 according to the instructions. For AIM2 inflammasome activation, macrophages were primed with Pam3CSK4 (100 ng mL^−1^) for 6h and transfected with Poly (dA:dT)(1 µg mL^−1^) using Lipofectamine 3000. After stimulation, cell culture supernatant was collected for ELISA assay of IL‐β (88‐7013, Invitrogen), TNF‐α (88‐7324, Invitrogen), IL‐6 (88‐7064, Invitrogen), and LDH (C0016, Beyotime Biotechnology) according to the manufacturer's instructions. Inhibitors for GSK461364 were added half an hour before LPS priming.

### Western Blot

Cell lysates were prepared by using cell lysis buffer (CST, 9803) supplemented with protease and phosphatase inhibitors. Protein samples were separated by 10% or 15% SDS‐PAGE and transferred onto PVDF membranes (Merck Millipore, ISEQ00010). The concentration of primary antibodies was according to the manufacturer's recommendation.

### Immunoprecipitation

Macrophage cells were lysed by cold IP buffer containing 50 mm Tris HCl (pH 7.4), 50 mm EDTA,200 mm NaCl, and 1% NP‐40 with protease and phosphatase inhibitors. The primary antibodies were incubated with whole cell lysates overnight at 4 °C to form the antigen‐antibody complex. Next morning, the antigen‐antibody complex was incubated with protein A/G‐agarose beads (Santa Cruz, sc‐2003) for 2 h at 4 °C. After washing with IP buffer three times, the beads were eluted in loading buffer and subjected to immunoblotting analysis. HEK293T cells were transfected with expression plasmids using Linear Polyethylenimine, according to the manufacturer's protocol. 24 h after transfection, cells were lysed with ice‐cold IP buffer. The cell lysates were incubated for 2 h at 4 °C with Anti‐Flag affinity gel (Sigma, A2220)or Pierce TM Anti‐c‐Myc Agarose (Thermo Fisher Scientific,20 168). The gels were washed five times with IP buffer, and the beads were eluted in loading buffer and subjected to immunoblotting analysis.

### Plasmids and Transfection

NLRP3, caspase‐1, pro‐IL‐1β, ASC, and NEK7 were described earlier.^[^ ^26^ ^]^ PLK1 full‐length sequences were obtained from mouse peritoneal macrophage cDNA, then cloned into the pcDNA3.1 vector that contained different tags. All constructs were confirmed by DNA sequencing. The primers were as follows: 3′. PLK1 forward, 5′‐AACGGGCCCTCTAGACTCGAGATGAATGCAGCGGCCAAAG‐3′, PLK1 reverse, 5′‐TAGTCCAGTGTGGTGGAATTCGGAGGCCTTGAGGCGGTTG‐3′. Plasmids were transiently transfected into HEK293T cells with Linear Polyethylenimine according to the manufacturer.

### Reconstitution of NLRP3 Inflammasome in HEK293T Cells

The reconstitution system in HEK293T cells was according to the previous study.^[^ ^41^ ^]^ In brief, the HEK293T cells were seeded into 24‐well plates in DMEM medium. The following morning, cells were transfected with plasmids expressing (ASC, 25 ng, pro IL‐1β, 250 ng, NLRP3, 25 ng, pro‐caspase‐1, 5 ng, NEK7, 200 ng, or NEK7 mutants, 200 ng) and PLK1, 100ng. After 24h, the medium was replaced and Nigericin (10 mm) was added to the medium after 5h.1h later, supernatant was harvested. IL‐1β secretion was analyzed by ELISA.

### siRNA‐Mediated Gene Silences in Mouse Primary Peritoneal Macrophages

Silencing the expression of mouse endogenous genes in mouse peritoneal macrophages was achieved by a single gene‐specific siRNA (Sangon Biotech). The sequences of siRNA used in this study were as follows:si‐PLK1, 5′‐ GGUAUCAGCUGUGUGACAATT‐3′. The scrambled negative control siRNA was 5′‐ UUC UCC GAA CGU GUC ACG U‐3′. Transfection of siRNAs was performed 48 h after plating using the Lipofectamine RNAiMAX Transfection Reagent (Invitrogen, 13 778) according to the manufacturer's instructions.

### Immunofluorescence Assay

Mouse macrophages were plated on glass coverslips (3 × 10^4^ cells per well) overnight, and transfection of siRNAs, inhibitor, and stimulation was performed as before. After stimulation, cells on coverslips were washed three times with PBS, fixed with 4% paraformaldehyde, pH 7.4, at room temperature for 10 min, and permeabilized with 0.15% Triton X for 10 min. The cells were incubated with the indicated primary antibodies (dilution in PBS containing 3% BSA) overnight at 4 °C, followed by staining with fluorochrome‐conjugated secondary antibodies (1:50 in PBS containing 3% BSA). Nuclei were co‐stained with DAPI. Fluorescence in cells was detected by a Fluorescence microscope (Nikon Ti2‐U), and at least 100 cells were counted in each slide.

### ASC Oligomerization Assay

Mouse macrophages were washed in PBS and lysed by Triton buffer containing 50 mm Tris, pH 7.5, 150 mm NaCl, 0.5% Triton X‐100, Cocktail. Then centrifuged at 6000×g for 15 min at 4 °C. The soluble supernatant was collected and boiled for 10min at 100 °C after adding 5×loading buffer. The pellets were washed twice with Triton buffer and dissolved in Triton buffer. 10 mm disuccinimydylsuberate was added to the resuspended pellets, which were incubated for 30 min at 37 °C. Samples were then centrifuged at 6000×g for 10 min at 4 °C. The supernatants were removed, and the cross‐linked pellets were resuspended in 30 µL of 2×loading buffer. Samples were boiled for 10 min at 100 °C and analyzed by Western blot.

### ASC Speck Formation

Primary peritoneal macrophage cells were seeded on chamber slides overnight. The next day, the cells were treated with the indicated stimuli. Next, the cells were washed three times with PBS buffer, fixed in 4% paraformaldehyde (PFA) for 10 min, permeabilized with 0.1% Triton X‐100 for 15 min, and blocked with PBS buffer containing 3% BSA. Cells were then stained with anti‐ASC (1:200) at 4 °C for 12 h and with DyLight 488–labeled secondary antibody (1:50) at room temperature for 60 min. Last, DAPI was used to stain nuclei. Cells and tissues were visualized using a fluorescence microscope (Nikon Ti2‐U).

### Cellular Thermal Shift Assay (CETSA)

Peritoneal macrophages were collected and resolved in RlPA buffer containing the protease inhibitor cocktail. The cell lysates were centrifuged at 12 000xg for 15 min at 4 °C, after which the supernatants were collected. The lysate supernatants were incubated with GSK461364 (10 µm) or the control (DMSO) for 1h at room temperature. Subsequently, each drug‐treated cell lysate was aliquoted into PCR tubes (60 µL each), and the tubes were heated at different temperatures (37, 47, 57, 67, 77, and 87 °C) for 5 min in the PCR apparatus, followed by cooling at room temperature for 3 min. The soluble fractions were separated after centrifugation of the lysates at 20000 × g for 20 min at 4 °C, transferred to new microcentrifuge tubes, and followed by western blotting with anti‐NLRP3 and anti‐PLK1 antibodies.

### Surface Plasmon Resonance Experiment

3.1 Chip Preparation The activator was prepared by mixing 400 mm EDC and 100 mm NHS immediately prior to injection. The CM5 sensor chip was activated for 420 s with the mixture at a flow rate of 10 µL min^−1^. Dilute PLK1 to 50 µg mL^−1^ in immobilization buffer, then injected into the sample channel (Fc2) at a flow rate of 10 µL min^−1^, which typically results in immobilization levels of 10 200 RU. The reference channel (Fc1) does not need a ligand immobilization step. The chip was deactivated by 1 m Ethanolamine hydrochloride at a flow rate of 10 µL min^−1^ for 420 s. Dilute GSK461364 with the same analyte buffer to 6 concentrations (25, 12.5, 6.25, 3.12, 1.56, and 0 µm). GSK461364 was injected into channel Fc1‐Fc2 at a flow rate of 30 µL min^−1^ for an association phase of 120 s, followed by 200 s dissociation. The association and dissociation processes were all handled in the analyte buffer. Repeat 6 cycles of analyte according to the analyte concentrations in ascending order. After each cycle of interaction analysis, the analyte will dissociate naturally. The chip doesn't need to be regenerated.

### GST Pulldown Assay

Recombinant mouse NEK7‐GST and PLK1‐his proteins were purchased from Sino Biological (Beijing, China), which were expressed by the eukaryotic expression system. Glutathione resin‐immobilized GST or NEK7‐GST protein were incubated with GST beads on a rotator in GST pull down buffer (20 mm Tris‐HCl pH7.9, 150 mm NaCl, 0.5 mm EDTA, 10% glycerol, 1 mm DTT, 0.1% Triton X‐100) for half an hour at 4 °C. Beads were washed 3 times with GST pull down buffer and incubated with PLK1‐his protein for 2 h at 4 °C.Beads were washed 3 times with GST pull down buffer and analyzed by immunoblot.

### Phos‐Tag Gel

The Phos‐tag reagent(F4002) was purchased from APExBio. To analyze the phosphorylation profiles of NEK7 by immunoblot, 50 µm Phos‐tag was added to the 6% SDS‐PAGE gels.

### In Vitro Kinase Assays

PLK1‐his proteins, NEK7‐GST protein, were subjected to the kinase buffer(9802, CST) in the presence of 200 µm ATP(9804, CST)or without. The reaction mixtures were incubated for 60 min at 30 °C, stopped with 5x loading buffer, heated to 99 °C for 10min, and subjected to SDS‐PAGE analysis in the presence of 50 µm Phos tag.

### LPS‐Induced Endotoxemia

Wild‐type C57BL/6 male mice (8‐week‐old) were intraperitoneally injected with saline or GSK461364 (10 and 25 mg kg^−1^) half an hour before mice were injected with LPS (20 mg kg^−1^ body weight) for 8 h. The serum levels of IL‐1β, IL‐6, and TNF‐α were measured by ELISA. For histology, paraffin‐embedded lung tissue sections were stained with H&E.

### DSS‐Induced Colitis

For acute experimental colitis induction, C57BL/6 mice were treated with 3% DSS or saline in their drinking water for 7 days. During the experiment, body weight, stool, and body posture were monitored daily to assess the DAI.^[^ ^42^ ^]^ The DAI was the combined score of weight loss compared with initial weight, stool consistency, and bleeding. The details were as follows: a) weight loss (0 point = none, 1 point = 1–5% weight loss, 2 points = 5–10% weight loss, 3 points = 10–15% weight loss, and 4 points = more than 15% weight loss), b) stool consistency or diarrhea (0 point = normal, 2 points = loose stools, and 4 points = watery diarrhea), and c) bleeding (0 point = no bleeding, 2 points = slight bleeding, and 4 points = gross bleeding). Mice were euthanized at the indicated time points, and the colon was immediately collected for colon length measurement and histological analysis.

### Proximity Ligation Assay (PLA)

Proximity ligation assays were performed using Duolink reagents (Sigma) following the manufacturer's instructions to visualize the interaction between PLK1 and NEK7 proteins. Cells were first fixed, permeabilized, and blocked, then incubated overnight at 4 °C with primary antibodies. After primary antibody incubation, cells were exposed to a combination of the corresponding PLA probes and secondary antibodies conjugated to oligonucleotides for 1 h at 37 °C. Following a wash with buffer A [0.01 m Tris‐HCl (pH 7.4), 0.15 m NaCl, 0.05% Tween 20], cells were incubated with ligation mix (Sigma, DUO92008) for 30 min at 37 °C, allowing the formation of a closed circle DNA template when the PLA probes were in close proximity. After another wash with buffer A, cells were incubated with polymerase mix (Sigma, DUO92008) for 100 min at 37 °C to allow rolling circle amplification. Sequential washes with buffer B [0.2 m Tris‐HCl (pH 7.4), 0.1 m NaCl] and buffer C (a 10‐fold dilution of buffer B in water) were performed before mounting coverslips (Citotest, 10 212 450 C) onto microscopy slides. Images were acquired using a 63×/1.4 oil immersion objective on a Leica STELLARIS 5 confocal microscope (Leica).

### Statistical Analysis

All statistical analyses were performed with GraphPad Prism software. Independent sample *t*‐tests and two‐way ANOVA followed by the Bonferroni correction were performed in this study. *P‐value* <0.05 was considered statistically significant, and the details of the statistical analyses can be found in the figure legends.

### Ethics Statement

The animal experiments were approved by the Institutional Animal Care and Use Committee of Central South University (APU‐2025‐0019).

## Conflict of Interest

The authors declare no conflict of interest.

## Author Contributions

R.L., M.M., D.W., and L.L. contributed equally to this work. K.Z. supervised the whole project, K.Z. designed the research, K.Z. R.L. and M.M. wrote the manuscript, R.L., M.M., D.W., and L.L. performed the experiments, analyzed the data, and made the figures. X.X., F.W., L.H., X.L., and G.K. provided technical help, H.L. and R.N. helped with data analyses and discussions, Q.Z. and S.W. assisted in data interpretation and edited the manuscript.

## Supporting information



Supporting Information

## Data Availability

The data that support the findings of this study are available in the supplementary material of this article.
