# Supplementary material for: GSK461364 Inhibits NLRP3 Inflammasome by Targeting NEK7 Phosphorylation
Source: Adv Sci (Weinh). 2025 Sep 15;12(44):e04816. doi: 10.1002/advs.202504816 (PMC12667451; doi:10.1002/advs.202504816)

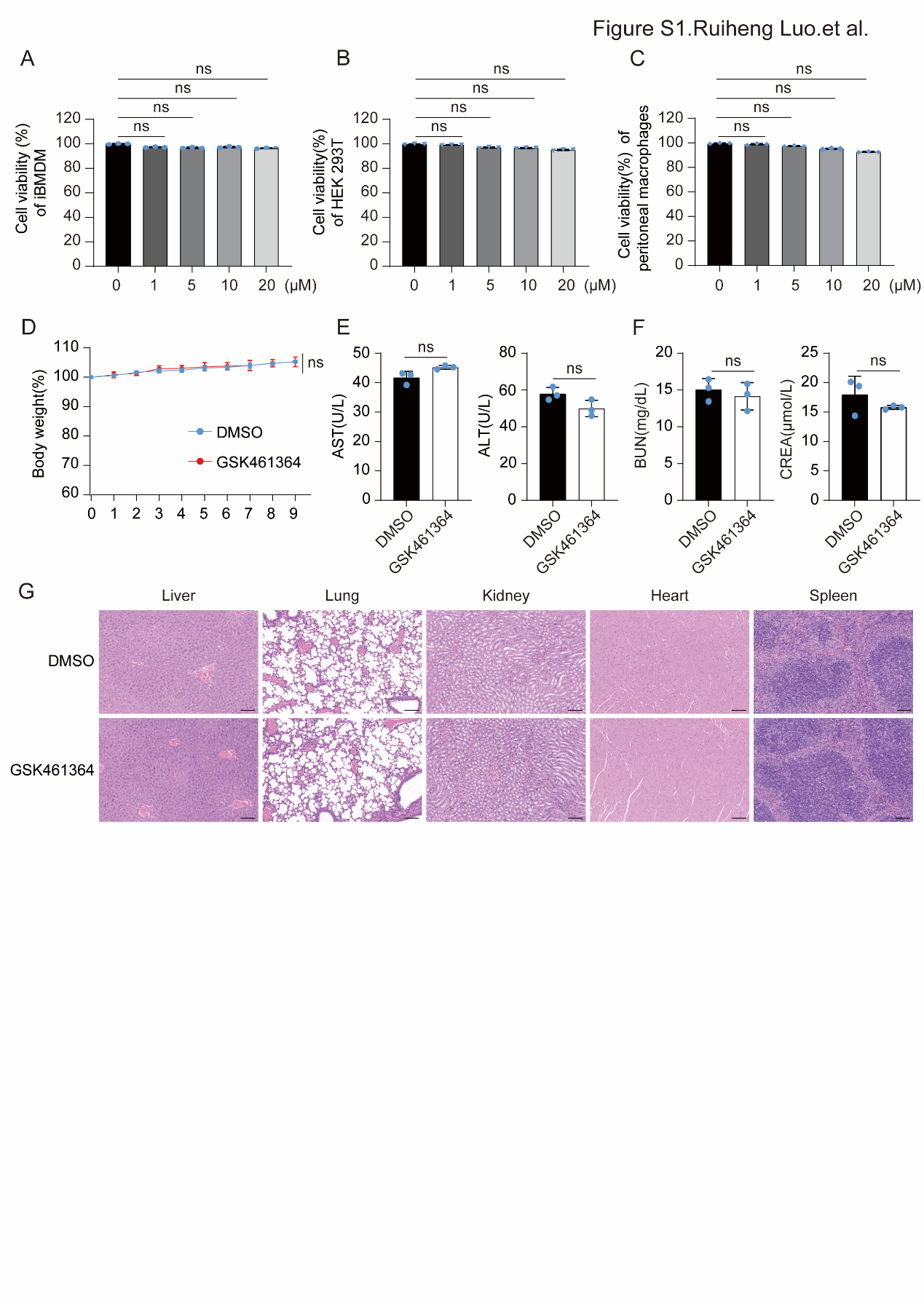


Figure S1. Evaluation of GSK461364 safety in vitro and in vivo.

（A-C）Cell viability of iBMDMs, HEK293T cells, and mouse primary peritoneal macrophages following 1 hour treatment with indicated concentrations of GSK461364.

（D）Body weight monitoring of mice during GSK461364 treatment.

（E）Serum levels of ALT and AST.

（F）Serum levels of CREA and BUN.

（G）Representative H&E staining of major organs harvested 9 days post-GSK461364 treatment. Scale bar, 100 μM.

Results are represented as mean ± SD and typical photographs are representative of three biological independent experiments with similar results. Statistical analyses were carried out via two-way ANOVA with the Bonferroni test for (A-F). ns P > 0.05.


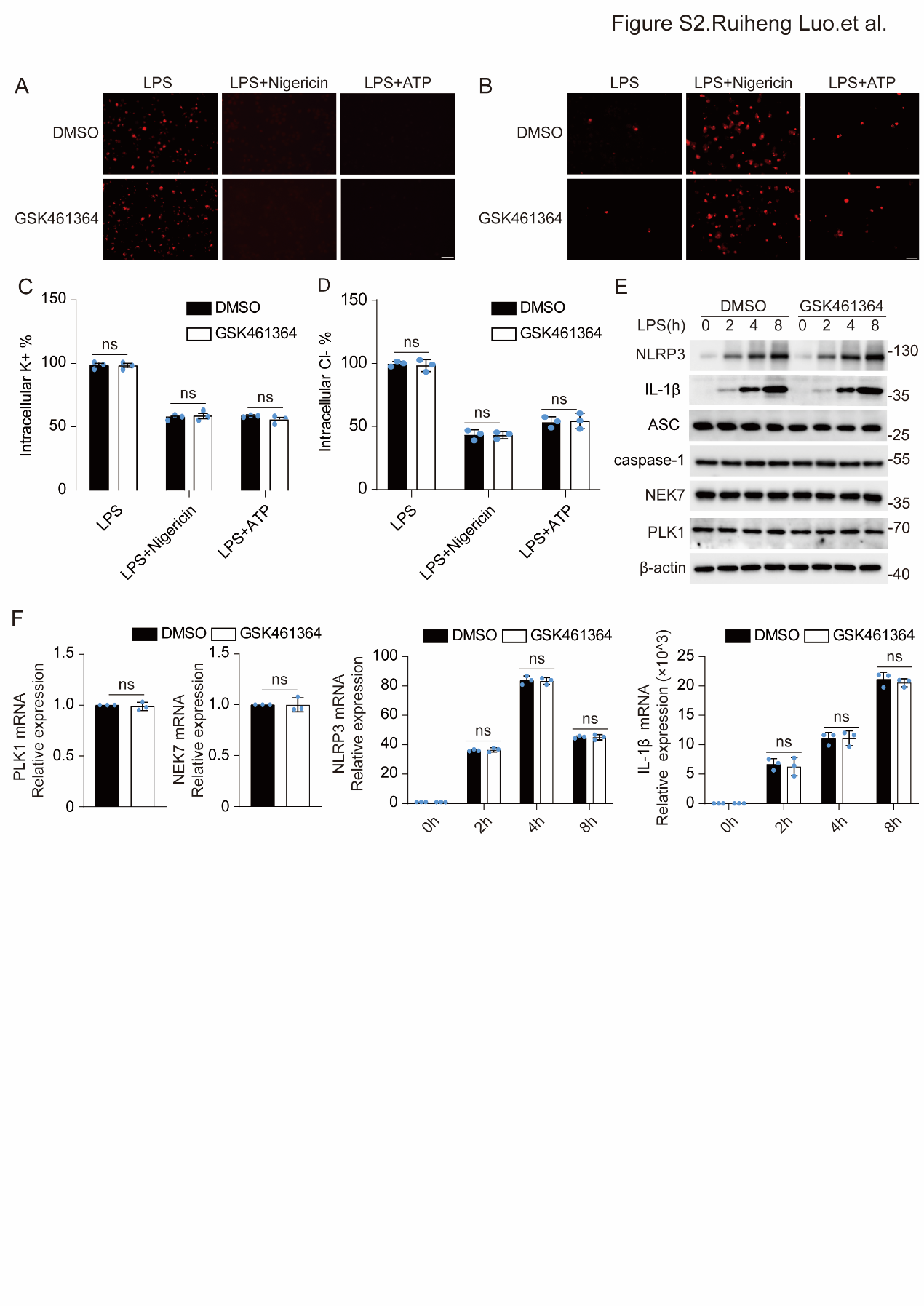


Figure S2. GSK461364 has no effects on mitochondrial injury and intracellular ion concentration.

（A-D）LPS-primed peritoneal macrophages from wild-type C57BL/6 were treated or untreated with 10 μM GSK461364, followed by stimulation with nigericin or ATP.

（A-B）Representative fluorescence images showing mitochondrial membrane potential (TMRM staining, A) and mitochondrial ROS production (MitoSOX staining, B) during NLRP3 inflammasome activation. Scale bar: 100 μm.

（C-D）Qualification of potassium(C) and chlorine(D) ion concentration during NLRP3 inflammasome activation. The results shown were representative of three independent experiments.

（E）Immunoblot analysis of protein expression in LPS-stimulated peritoneal macrophages at indicated time points.

（F）Quantitative real-time PCR analysis of NLRP3, pro-IL-1β, PLK1, and NEK7 mRNA expression levels in LPS-stimulated peritoneal macrophages.

Results are represented as mean ± SD , and typical photographs are representative of three biological independent experiments with similar results. Statistical analyses were carried out via two-way ANOVA with the Bonferroni test for (C-D,F). ns P > 0.05.


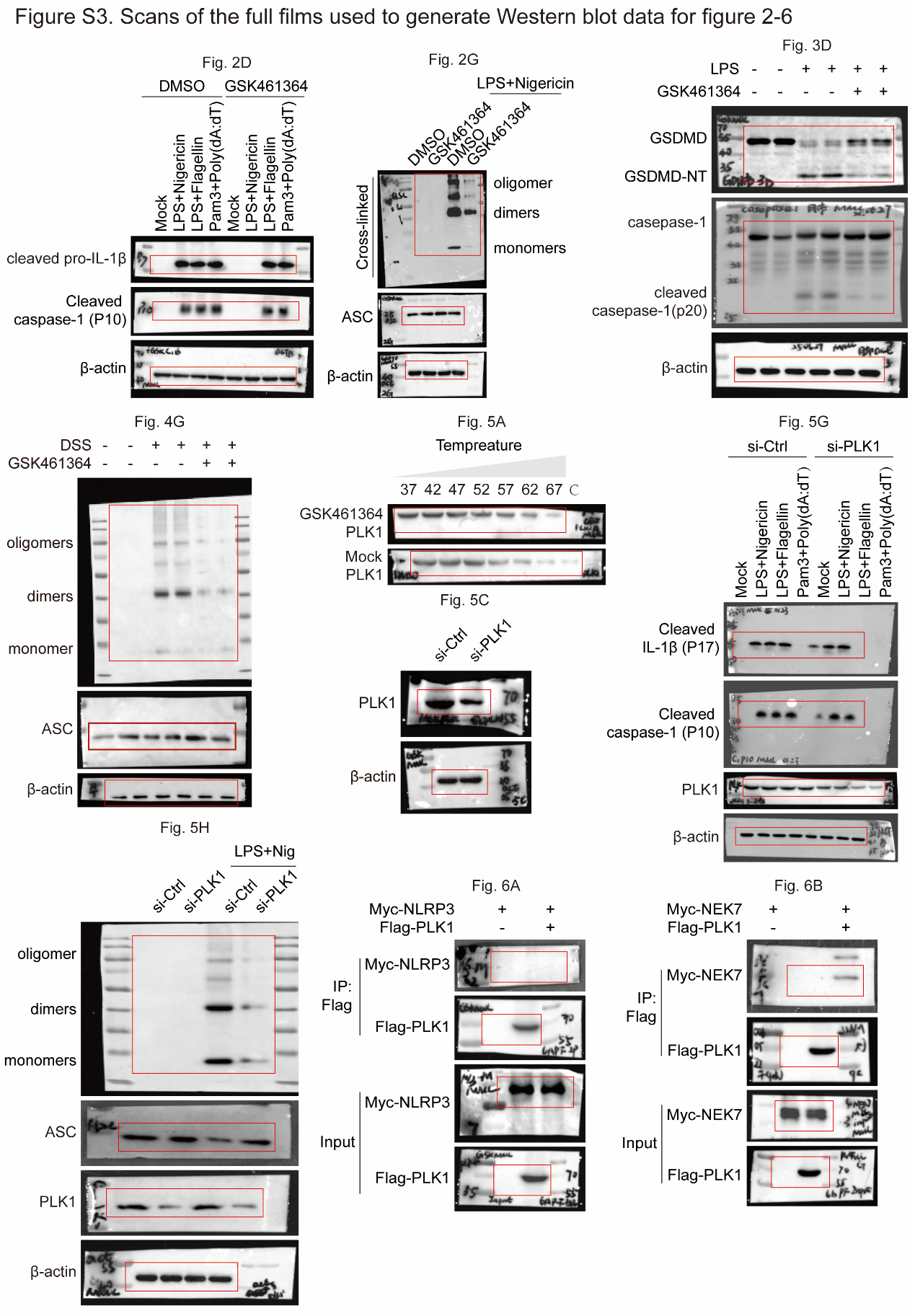

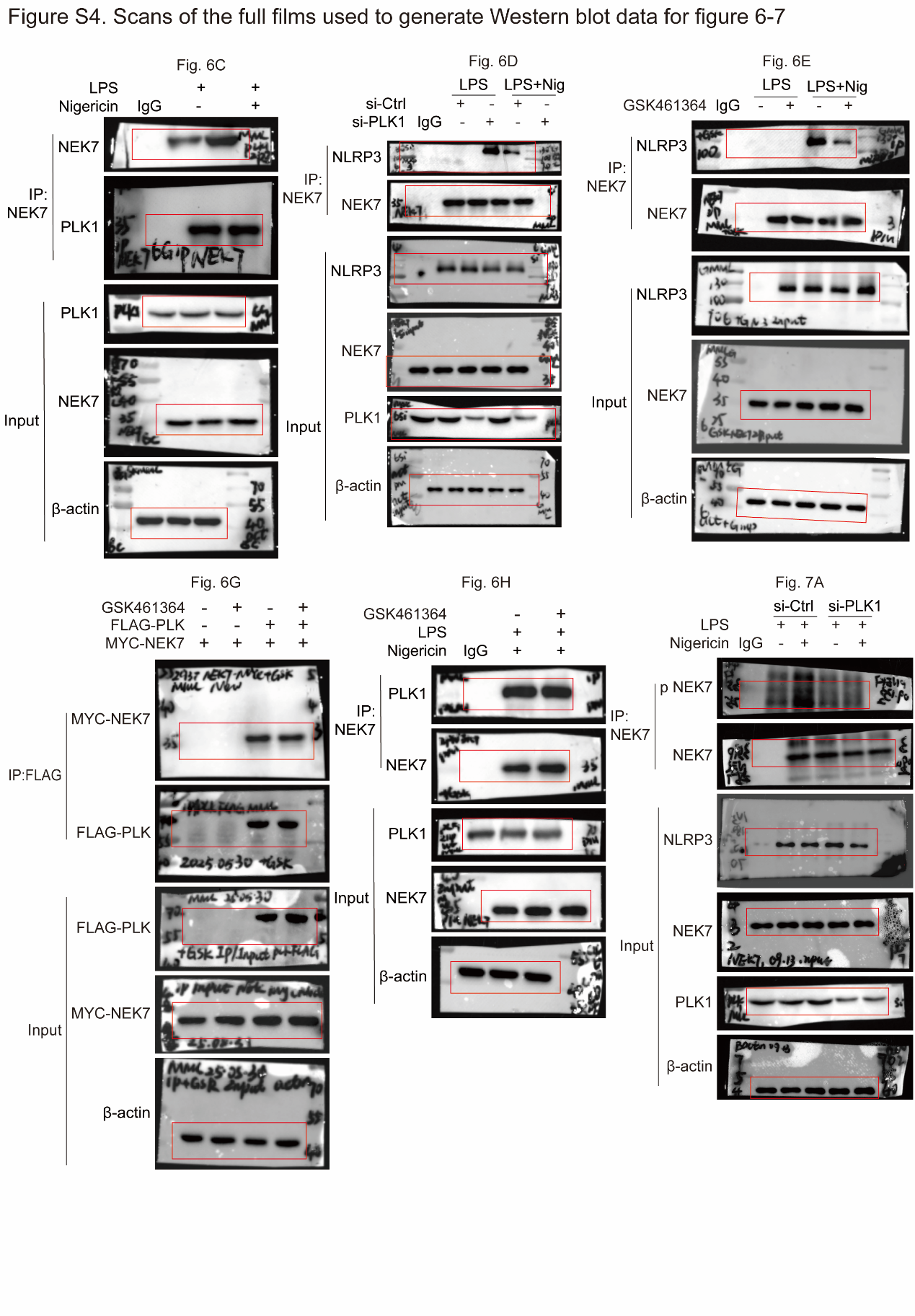

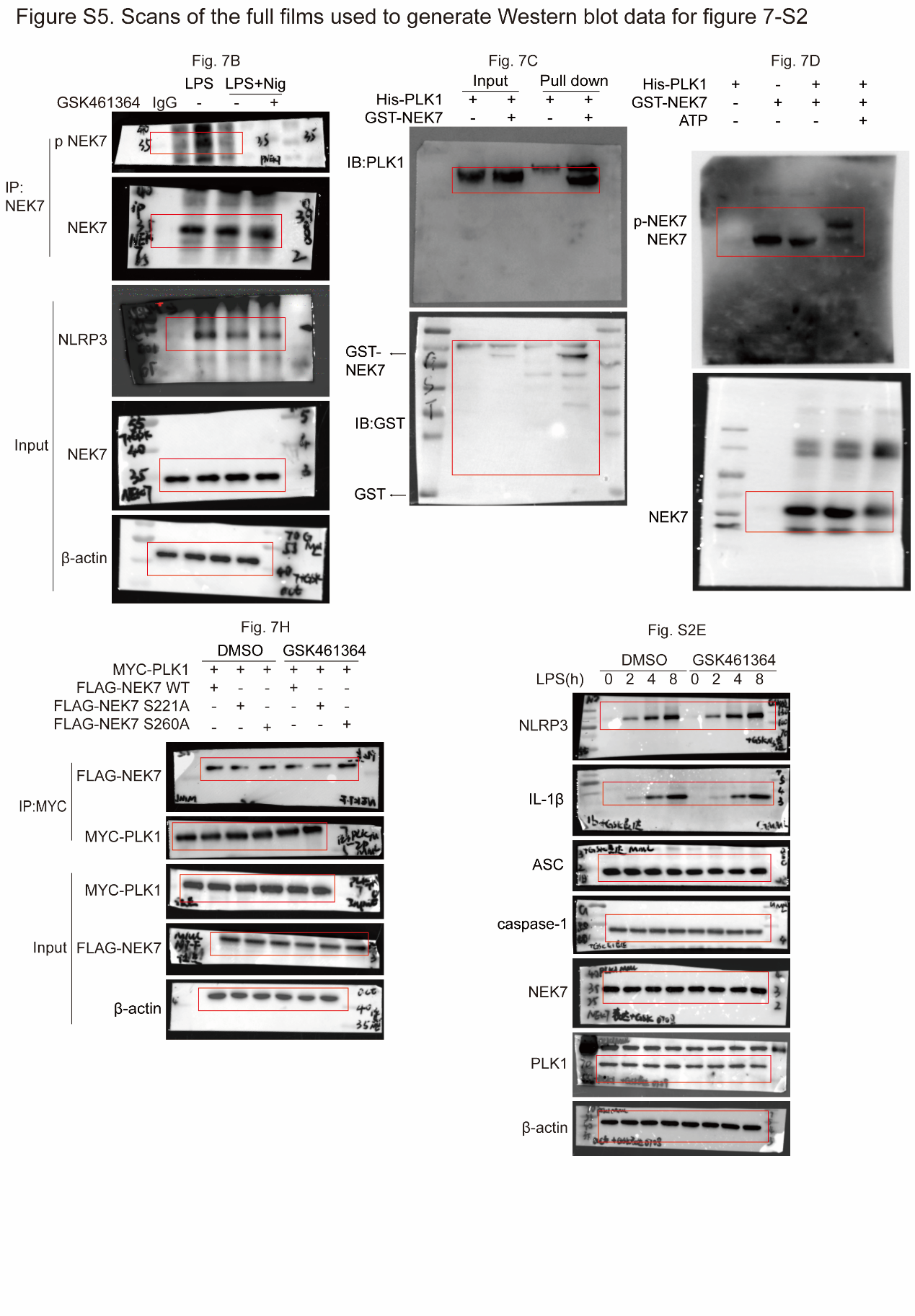

Supplement: Supplementary file 1 — Supporting Information [file ADVS-12-e04816-s001.docx]
